# A novel loss-of-function *SYCP2* variant causes asthenoteratozoospermia in infertile males

**DOI:** 10.3389/fgene.2025.1595720

**Published:** 2025-05-13

**Authors:** Cong Liu, Yinfeng Zhang, Youming Zhao, Haining Luo

**Affiliations:** ^1^ Department of Center for Reproductive Medicine, Tianjin Central Hospital of Gynaecology Obsterics, Tianjin, China; ^2^ Tianjin Institute of Gynaecology Obsteric, Tianjin Central Hospital of Gynaecology Obsterics, Tianjin, China; ^3^ Tianjin Key Laboratory of Human Development and Reproductive Regulation, Tianjin Central Hospital of Gynaecology Obsterics, Tianjin, China

**Keywords:** male infertility, whole exome sequence, *SYCP2*, gene variation, mini-gene splicing assay

## Abstract

**Background:**

Infertility is a multiplex disorder in the reproductive system. Unexplained infertility affects 2%-3% of reproductive-aged couples. Male factors contribute to about half of all infertility cases. About 15% of these cases are predicted to have a genetic etiology. With the wide application of whole exome sequencing (WES), more and more variations in male infertility have been identified.

**Methods:**

A patient diagnosed with asthenoteratozoospermia was involved in this study. WES was performed in the patient, and Sanger sequencing was used to confirm the variation. Mini-gene splicing assays were performed to validate the effect on the alternative splicing of the variation.

**Results:**

A novel heterozygous splice variant was identified in SYCP2 (c.2600+ 5G>C) in the patient ,which inherited from his phenotypically normal mother. SYCP2 encodes a protein critical for the synapsis of homologous chromosomes during meiosis I, and its disruption can impair spermatogenesis. Mini-gene splicing assays confirmed that this splicing variant impacted alternative splicing and that the stop codon appeared early, which was very likely to result in the loss of function of the protein and lead to the occurrence of male infertility.

**Conclusion:**

Our results suggested that the c.2600+5G>C variation in SYCP2 might be the genetic etiology for male infertility in this pedigree. This finding expanded the known genotype spectrum of male infertility and provided new etiological information for male infertility.

## 1 Introduction

Infertility is an important health problem with a multifactorial etiology that affects approximately 15% of couples who attempt pregnancy globally (Tamrakar and Bastakoti, 2019). In approximately 50% of these couples, a male factor plays an important role, which may exist either alone or in combination with female factors (Xie et al., 2018). Male infertility is a multifactorial pathological condition affecting approximately 7% of the male population (Li et al., 2024). The genetic factor of male infertility is highly complex, as testis and semen histological phenotypes are extremely heterogeneous, and at least 2000 genes are involved in spermatogenesis (Krausz and Riera-Escamilla, 2018). Understanding the genetic etiology of male infertility can provide genetic counselling and subsequent therapeutic interventions to patients, such as intracytoplasmic sperm injection (ICSI) and *in vitro* fertilization (IVF), as well as seeking donor sperm or adoption (Li et al., 2024). Therefore, identifying genetic variants associated with male infertility can provide patients with meaningful and actionable information.

The identification of novel candidate genes in infertile males has increased rapidly since the implementation of next-generation sequencing, including whole genome sequencing (WGS) and whole exome sequencing (WES). The molecular diagnostic project has become an important mean of clinical diagnosis. However, many genes have not yet accumulated sufficient evidence to be confidently implicated in male infertility.

*Synaptonemal complex protein 2 (SYCP2)* is a novel candidate gene associated with autosomal dominant male infertility and is located at 20q13.33 (Schilit et al., 2020). *SYCP2* encodes synaptonemal complex protein 2, an axial element in the proteinaceous synaptonemal complex (SC) (Schalk et al., 1999). SC assembly contributes to the pairing and segregation of homologous chromosomes during meiosis (Page and Hawley, 2003). *SYCP2* is important for spermatogenesis (Takemoto et al., 2020).

This study investigated the genetic cause of male infertility in a Chinese patient. WES and subsequent Sanger sequencing revealed a novel heterozygous variant in *SYCP2* (c.2600 + 5G>C). This variation was inherited from the patient’s healthy mother. The variation was verified to impact the alternative splicing of *SYCP2* and introduced an early stop codon that resulted in the prematuration and loss of function of *SYCP2*.

## 2 Materials and methods

### 2.1 Patients

A family with a proband diagnosed with male infertility was involved in this study. The family members who participated in this study were thoroughly informed about this study. This study was approved by the medical ethics committee of Tianjin Central Hospital of Gynecology Obstetrics (No. ZY2023001).

### 2.2 WES, variant interpretation, and sanger sequencing

Genomic DNA (gDNA) was isolated from peripheral blood via the DNeasy Blood and Tissue Kit (QIAGEN, Germany). WES was performed via the sequencing platform of the Beijing Genomics Institute (BGI). All steps were performed according to the manufacturer’s instructions. Exomes were hybridized and captured by the xGen Exome Research Panel of Integrated DNA Technologies (IDT, America) and sequenced on the MGI-2000. The average sequencing depth was 100x‒150x, and the data quality was Q20 ≥ 90% and Q30 ≥ 90%. The original data obtained by sequencing were filtered through fastp software (Chen et al., 2018) and then passed through BWA software (Houtgast et al., 2018). The sequenced reads were collected, filtered for quality, and aligned to the human reference genome (hg19/GRCh37). The sequenced variants were annotated via ANNOVAR software (Wang et al., 2010)^,^ and mutations were screened on the basis of patients’ clinical information, population databases, disease databases, and bioinformatic prediction tools. Candidate pathogenic variants were scored in accordance with the criteria set by the American College of Medical Genetics and Genomics (ACMG) (Richards et al., 2015) and confirmed by Sanger sequencing.

### 2.3 Mini-gene construction

To construct the mini gene, we amplified the *SYCP2* fragment from the sectional intron26 (627 bp) to the sectional intron27 (84 bp) via nested polymerase chain reaction (PCR) with a primer pair and added the endonuclease recognition sequences of KpnI and XhoI to the front and end of the fragment, respectively. The detailed methods were presented in the supplementary materials. The PCR products were purified via alcohol and digested along with the vector pcMINI via the endonucleases KpnI and XhoI (New England Biolabs, America). The digested PCR products and vectors were purified via electrophoresis and ligated together with T4 ligase (New England Biolabs, America). Ligase products were transformed into DH5α competent cells, which were subsequently plated on LB plates coated with ampicillin. Single clones were then selected for proliferation and Sanger sequencing. The identified colonies were amplified, and plasmid DNA without endotoxin was extracted via the Rapid Plasmid Mini Kit (Simgen, China). The primers used in this study are listed in the Supplementary Material.

### 2.4 Cell transfection

Human embryonic kidney 293T (HEK293T) cells and HeLa cells were cultured in DMEM/high glucose (Gibco, America) supplemented with 10% FBS (Sigma, America) in a 37°C constant-temperature water bath incubator at 5% CO_2_. The cells were dissociated into single cells using trypsin-EDTA (Thermo Fisher, America) after they reached 80% confluence. The cells were counted, and 4 × 10^5^ cells were seeded in 6-well plates 24 h before transfection. The cell medium was changed to Opti-MEM (Thermo Fisher, America) 2 h before transfection. Lipofectamine 2000 (Thermo Fisher, America) was incubated for 20 min at room temperature, the mixture was mixed thoroughly with 1 μg of plasmid, and then, the mixture was gently added to the cell medium. The cell medium was replaced with fresh culture medium supplemented with 10% FBS 12 h after transfection.

### 2.5 RT-PCR and sanger sequencing

Total RNA was extracted 48 h after transfection via TRIzol reagent (TaKaRa, China) following routine procedures. Reverse transcription‒polymerase chain reaction (RT-PCR) was performed via ABScript III RT Master Mix for qPCR with gDNA Remover (ABclonal, China) according to the manufacturer’s instructions. cDNA was used as a template for PCR with the primer pair F/R (Supplementary Material). The amplification products were subjected to electrophoresis on a 1% agarose gel and Sanger sequencing.

### 2.6 Bioinformatics analyses

The *SYCP2* gene sequence was obtained from the NCBI Gene database (https://www.ncbi.nlm.nih.gov/gene/). The molecular structure of the cryo-EM structure of GATOR1 was viewed with Mol* View (Sehnal et al., 2021) and stored in the RCSB PDB (Berman et al., 2000).

## 3 Results

### 3.1 Clinical report for the patient

We sought to identify the genetic etiology of infertility for a male research participant who presented with a 2-year history of infertility at age 30. His assessment indicated asthenoteratozoospermia (AT) (the semen test results were shown in Table 1) in accordance with the WHO Laboratory Manual for the Examination and Handling of Human Semen, fifth edition. The patient displayed no dysmorphic features and had normal serum levels of follicle-stimulating hormone (FSH), luteinizing hormone (LH), and testosterone. Y chromosome microdeletions were normal. The couple pursued ICSI as a treatment for male infertility. However, the couple did not become pregnant after ICSI treatment at the center. Despite multiple attempts at ICSI and despite successful fertilization, the couple was unable to obtain viable embryos. Eventually, the patient discontinued the treatment.

**TABLE 1:** Patient semen characteristics.

Semen parameters	The patient in this study	Reference values
Total semen volume (mL)	1	>1.5
Concentration (10^6^/mL)	28	>15.0
the normal morphology rate	3	>4
Motility (%)	23	>40.0
Progressive motility (%)	13	>32.0

### 3.2 Genetic analysis identifies novel heterozygous *SYCP2* variants in the patient

**FIGURE 1:**
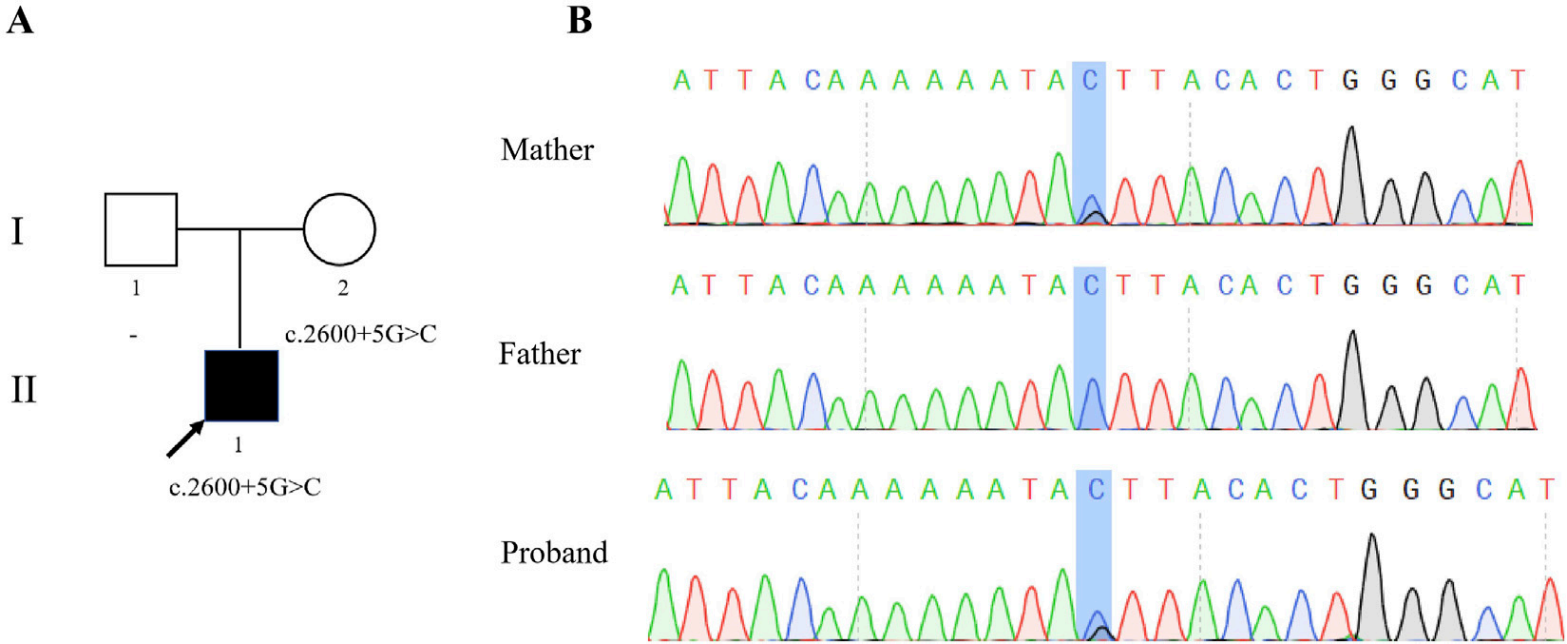
Identification of a heterozygous variation in *SYCP2*. **(A)** Pedigree of the family. The proband (II-1) was diagnosed with male infertility, as indicated by filled symbols with arrows in the pedigree. **(B)** Sanger sequencing of the proband and his parents revealed a c.2600 + 5G>C mutation in the *SYCP2* gene, which was maternally inherited.

To explore the genetic factors contributing to the infertility of the patient, WES and subsequent validation through Sanger sequencing were performed. The patient was found to have a heterozygous variant in intron 27 of *SYCP2*, NM_014258.4:c.2600 + 5G>C (Figure 1A). Sanger sequencing via the primer SYCP2-F/R confirmed that this variant was inherited from the patient’s mother, who had a normal phenotype (Figure 1B).

Notably, this variant was absent in several major population databases, including the 1KGP (1000 Genomes Project, Phase 3), ESP6500 (Genome Aggregation Database, V2), gnomAD (Genome Aggregation Database, r2.0.1) and ExAC (Exome Aggregation Consortium, r0.3.1) databases, highlighting its rarity within these populations. This is the first report of this phenomenon in this study. Bioinformatics analysis via “Ada” and “RF” scores predicted that this variant was likely to influence splicing (Supplementary Table S2). According to the ACMG guidelines, the novel variation c.2600 + 5G>C was designated as a variant of uncertain significance (VUS).

### 3.3 Functional splicing examination of the variant with mini-gene splicing assays

**FIGURE 2:**
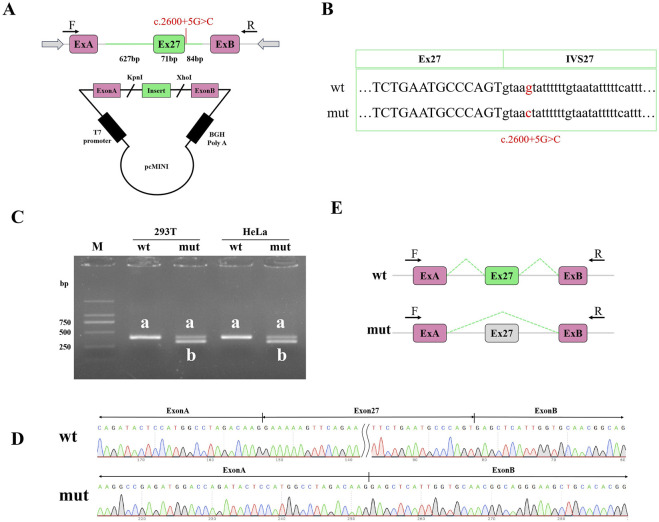
Examination of functional splicing of the variant with mini-gene splicing assays. **(A)** Schematic diagram of the constructed mini-gene. ExA and ExB are exonic sequences of the plasmid. **(B)** Sanger sequencing confirmed that the wild-type and mutant fragments were successfully introduced into the mini-gene construct. **(C)** RT-PCR was performed to verify alternative splicing in the wild-type and mutant groups. Abnormal splicing bands in the mutant groups were not detected in the HEK293T cells or the HeLa cells. Agarose gel electrophoresis revealed that the wt had only one band, and the labelled a and mut proteins produced two bands, labelled a and b. **(D)** The bands in a and b were identified via Sanger sequencing. Alternative splicing was affected by the c.2600 + 5G>C variation in SYCP2. PCR product sequencing revealed exon 27 skipping. **(E)** Alternative schematic diagram. wt, wild type; mut, mutant type; M, DL2000 DNA ladder.

To validate the effect of the c.2600 + 5G>C variation on RNA alternative splicing, we conducted a mine gene splicing assay by constructing containing the wild-type (wt) and mutation-type (mut) target DNA fragments (Figure 2A). The vectors were confirmed by Sanger sequencing (Figure 2B). Plasmid DNA without endotoxin was transfected into HEK293T cells and HeLa cells. The total RNA of the transfected cells was extracted, and reverse transcription was performed to obtain cDNA. Agarose electrophoresis of the RT-PCR products revealed two distinct splicing patterns (Figures 2C,D). The full uncropped Gels image presented in Supplementary Figure S2. Sanger sequencing revealed that abnormal splicing occurred in cells transfected with the mutation plasmid and that the mutation C.2600 + 5G>C affected the normal splicing of gene mRNA (Figure 2E). The detection results of pcMINI and PCMINI-C were consistent, as shown inSupplementary Figure S1. There was one abnormal transcript after mutation: exon 27 skipping. The mutation caused exon 27 skipping, which was expressed in the cDNA as c.2530_2600del. Exon 27 skipping caused a subsequent frameshift and produced an early stop codon in exon 28, which might produce a truncated protein 844 aa in length. Thus, the variation was described as SYCP2:c.2600 + 5G>C (p.Lys845*).

### 3.4 Translation analysis and protein modelling

**FIGURE 3:**
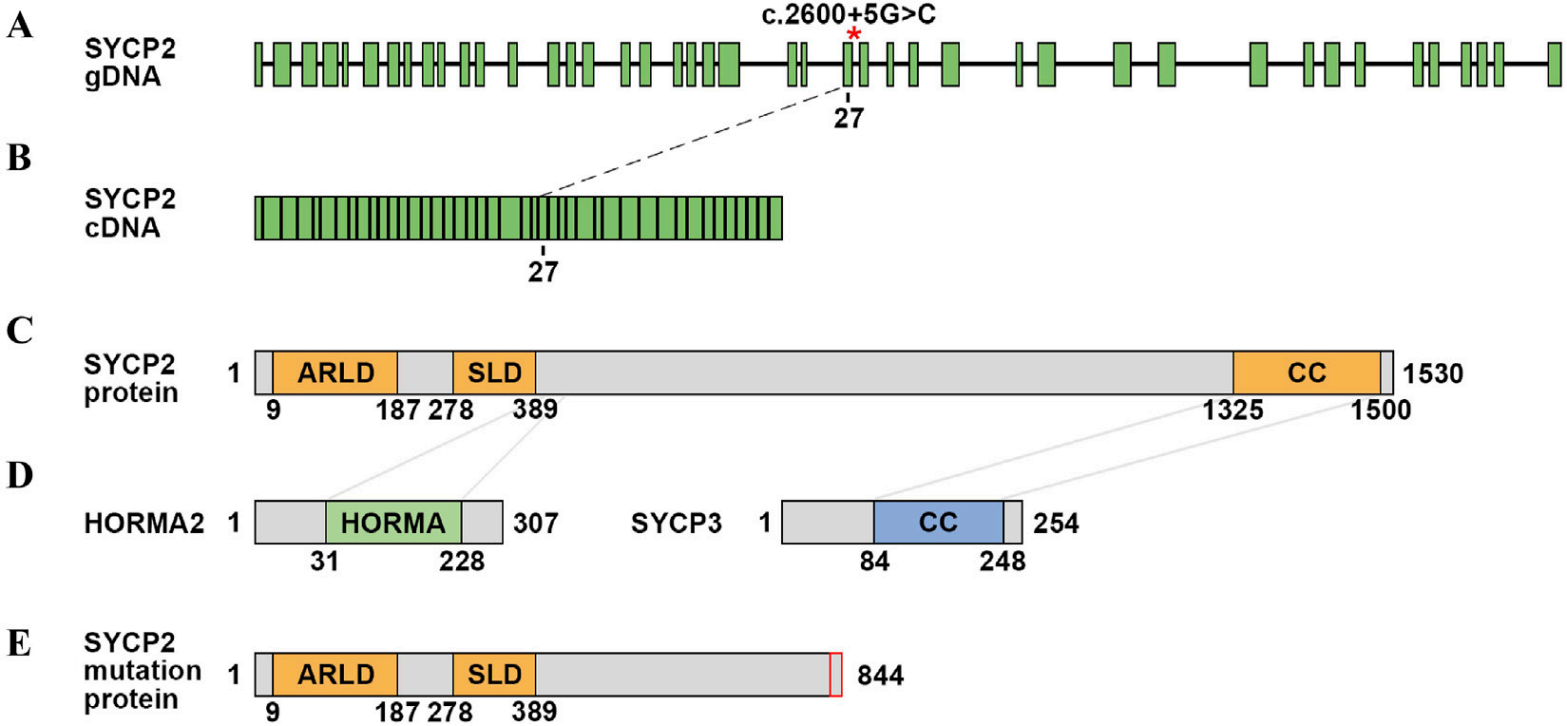
Schematic of the *SYCP2* gene and protein domain. **(A)** gDNA structure of *SYCP2*. The red asterisk represents the variation site. **(B)** cDNA structure of SYCP2. **(C)** Schematic diagram of the SYCP2 domain. The SYCP2 protein contains the ARLD domain, SLD domain and CC domain. **(D)** Schematic diagram of the HORMA and SYCP3 structures. The connected parts interact with the SYCP2 domain. **(E)** Schematic diagram of the predicted protein structure of mutated SYCP2.

We further analysed the cDNA sequence of *SYCP2* and found that the variation led to a frameshift and early appearance of the stop codon, which resulted in the prematuration of SYCP2 with 844 amino acid residues. Mutation of SYCP2 resulted in the loss of the whole coiled-coil (CC) domain (Figure 3). In rodents, SYCP2 directly interacts with SYCP1 and SYCP3 through its C-terminal domain and internal curly helix domain, respectively (Winkel et al., 2009). The deletion of the SYCP3-interacting domain of SYCP2 leads to severe defects in SC formation in mice, and males are sterile (Yang et al., 2006). The deletion of the CC domain meant that the protein was fundamentally changed and that its function was severely impaired.

## 4 Discussion

Many Mendelian disorders are genetically heterogeneous, with a multitude of different disease genes in which a variety of disease-causing variants have been discovered (Zhao et al., 2015). With the development of next-generation sequencing (NGS), an increasing number of disease-causing genes have been discovered. Accurate molecular diagnosis can provide an important basis for genetic counselling, specific treatment and family planning, and prognosis management (Ellingford et al., 2015).

Male infertility is a common disorder among reproductive-aged couples. Understanding the precise causes may directly inform therapies for infertile couples (Schilit, 2019). In this study, we described a patient with a clinical presentation compatible with male infertility and discovered a novel germline splicing variant, c.2600 + 5G>C, in *SYCP2*. Our experimental results revealed that this mutation caused exon 27 skipping, leading to subsequent changes in the reading frame. A premature termination codon (PTC) was generated within exon 28, and a truncated protein with a length of 844 aa was likely to be generated, destroying the curly helix region, which could form a heterodimer with the SYCP3 protein and play an important role in the assembly of the synaptic complex and the chromosome coupling process (Takemoto et al., 2020). Mice lacking the coiled-coil domain of *Sycp2* exhibit spermatocyte apoptosis and male-specific infertility (Yang et al., 2006). According to ACMG guidelines, the c.2600 + 5G>C variant was predicted to be likely pathogenic (LP), which was upgraded from VUS. The detection of the new mutation expanded the variation spectrum of *SYCP2* and provided the basis for the subsequent treatment of this family. However, surprisingly, the concentration of the patient’s semen was normal. We suspected that meiotic errors such as aneuploidy or DNA fragmentation due to SYCP2 dysfunction could lead to failed fertilization, even if sperm were present in this patient.

The penetrance of the disorder may be unknown due to the ascertainment of affected cases (Li et al., 2024). Indeed, there was an azoospermic proband with a homozygous loss-of-function variant in *SYCP2* whose father might have incomplete penetrance in the heterozygous state (Xu et al., 2023). Notably, in this study we found that female carrier was fertile, and maternally inherited SYCP2 variants had been seen in other reports (Li et al., 2024). In addition, disruption of Sycp2 gene has been shown to cause male infertility, but only female infertility in mouse model (Yang et al., 2006), suggesting that the relationship between SYCP2 and male infertility does not extend to female infertility. It was speculated that SYCP2 homologue SYCP2L may play an important role in female fertility (Li et al., 2024).

Mammalian RNA splicing is a delicate process whose precise coordination is not fully understood, but its regulation is critical for the proper expression of most genes and their isoforms (Zheng, 2004). Accurate pre-mRNA splicing is critical for proper protein translation and relies on the existence of consistent cis sequences that define exon‒intron boundaries and regulatory sequences recognized by splicing mechanisms (Wang et al., 2024). Variants that disrupt normal pre-mRNA splicing are increasingly recognized as major causes of monogenic disorders. Mutations in the canonical splice sequences usually lead to single-exon skipping, but the exact effect of specific splicing mutations on alternative pre-mRNA splicing needs further validation. Some nonclassical splicing events do not result in frameshifts, which might have a mild effect on protein function. In our study, the c.2600 + 5G>C variant in *SYCP2* causes the skipping of exon 27, as expected, and results in a frameshift and early appearance of a stop codon, causing the *SYCP2* protein to be prematurated and lose its normal function. The importance of understanding this process and being able to predict which variants alter splicing is therefore essential to understanding human disease.

*SYCP2* (OMIM 604105) is located on chromosome 20q13.33 and spans 70 kb in length. The *SYCP2* protein contains 1,530 amino acids. SYCP2 is a component of the synaptic complex and plays an important role in meiosis. At present, relatively few cases of *SYCP2* gene mutations have been reported, with the majority being loss-of-function mutations. In a recent study, three frameshift variants in *SYCP2* were identified in men with azoospermia, suggesting that heterozygous loss-of-function variants in *SYCP2* might be responsible for the low sperm count and subsequent infertility (Schilit et al., 2020). Substantial experimental evidence supports the role of *SYCP2* in male infertility, reinforcing the strong clinical validity of the classification of *SYCP2* as a gene associated with autosomal dominant male infertility (Li et al., 2024).

Nevertheless, there were also several limitations to this study. The results of the mini-gene splicing assay were not further validated in patient samples and we did not confirm the presence of the truncated protein in the variant by western blot. Moreover, histological analysis of the patient’s testicles was not performed.

## 5 Conclusion

In conclusion, in this article, we describe a proband with male infertility harboring a novel splicing variation, c.2600 + 5G>C (p.Lys845*), in *SYCP2* gene. The effect of this variation on alternative splicing and translation was confirmed by mini-gene splice assays. Our study provided a new source of evidence for the pathogenicity of splicing variation and expanded the phenotype and genotype spectrum of male infertility.

## Data Availability

The original contributions presented in the study are included in the article/Supplementary Material. The detailed original data is uploaded to figshare, DOI:10.6084/m9.figshare.28925081.
