# Supplementary material for: A novel loss-of-function SYCP2 variant causes asthenoteratozoospermia in infertile males
Source: Front Genet. 2025 May 13;16:1595720. doi: 10.3389/fgene.2025.1595720 (PMC12106479; doi:10.3389/fgene.2025.1595720)
Supplement: Supplementary file 1 [file Supplementaryfile1.docx]

**Supplementary Information**

**Table S1.** Primers for the sites used in the study.

| Name | Sequence (5’-3’) |
| --- | --- |
| SYCP2-F | CAGGAAAAAGTTCAGAAAAAAAGC |
| SYCP2-R | CTTACTCCAAGTTTTATGATAGGGTC |
| 39134-SYCP2-F | gcctccctggttcaagcaat |
| 39341-SYCP2-F | cccagccctaggaatgcaat |
| 40708-SYCP2-R | gtgaaggctttctaccccac |
| 40968-SYCP2-R | GACCTATCCGCACAAGCTTC |
| SYCP2-mut-F | TGTTACTTCTGAATGCCCAGTgtaaCtattttttgtaatatttttcatttt |
| SYCP2-mut-R | aaaatgaaaaatattacaaaaaataGttacACTGGGCATTCAGAAGTAACA |
| pcMINI-SYCP2-KpnI-F | ggtaGGTACCtactccttcctactcttcct |
| pcMINI-SYCP2-XhoI-R | tttcCTCGAGatgaagagcaatattagctc |
| pcMINI-C-SYCP2-KpnI-F | ggtaGGTACCggtatttgtctttctgtgtt |
| pcMINI-C-SYCP2-XhoI-R | TAGACTCGAGTCCAAGTTTTATGATAGGGT |
| F | ACTTAAGCTTatgagtgggctttggggtggccggtt |
| R | TAGAAGGCACAGTCGAGG |

**Table S2.** Clinical examination and variant information.

| gene | Mutation | ESP6500 | ExAC | 1000 genomes | gnomAD | dbscSNV_Ada_Score | dbscSNV_RF_Score |
| --- | --- | --- | --- | --- | --- | --- | --- |
| SYCP2 | c.2600+5G>C | NE | NE | NE | NE | 1.0000 | 0.934 |

Transcript, NM_014258.4; MAF, minor allele frequency; NE, not present.

**The detailed construction method of expression vectors**

Frist, two pairs of primers 39134-SYCP2-F and 40968-SYCP2-R, 39341-SYCP2-F and 40708-SYCP2-R were designed and nested PCR was performed using normal gDNA as the template. Then, using the product of the second round of nested PCR as the template and pcMINI-SYCP2-KpnI-F and pcMINI-SYCP2-XhoI-R as primers, the wild-type fragment was obtained. Using the product of the second round of nested PCR as the template and pcMINI-SYCP2-KpnI-F and SYCP2-mut-R as primers, the sectional mutation fragment was amplified. Using the product of the second round of nested PCR as the template and pcMINI-SYCP2-XhoI-R and SYCP2-mut-F as primers, another part mutation fragment was amplified. Finally, mixing these two mutated fragment in equal proportions as a template and pcMINI-SYCP2-KpnI-F and pcMINI-SYCP2-XhoI-R as primers, the complete mutation fragment was amplified. This step was also used for vector pcMINI-C. The steps using the primer pcMINI-SYCP2-KpnI-F were replaced with the primer pcMINI-C-SYCP2-KpnI-F and the steps using the primer pcMINI-SYCP2-XhoI-R were replaced with the primer pcMINI-C-SYCP2-XhoI-R.

**Results**

In order to exclude the effect of the vector on splicing, we further used the vector pcMINI-C to verify the mutation affected splicing. The experimental methods and operating procedures were the same as using the vector pcMINI-C. We conducted a mine-gene splicing assay by constructing expression vectors containing the wild-type (wt) and mutation-type (mut) target DNA fragments (FigureS1A). The vectors were confirmed by Sanger sequencing (FigureS1B). Plasmid DNA without endotoxin was transfected into HEK 293T cells and Hela cells. The total RNA of transfected cells was extracted, and reverse transcription was performed to achieve the cDNA. Agarose electrophoresis of RT-PCR products revealed two distinct splicing patterns (FigureS1C-S1D). Sanger sequencing uncovered abnormal splicing that occurred in cells transfected with the mutation plasmid and the mutation C.2600 +5G>C would affect the normal splicing of gene mRNA. (FigureS1E).


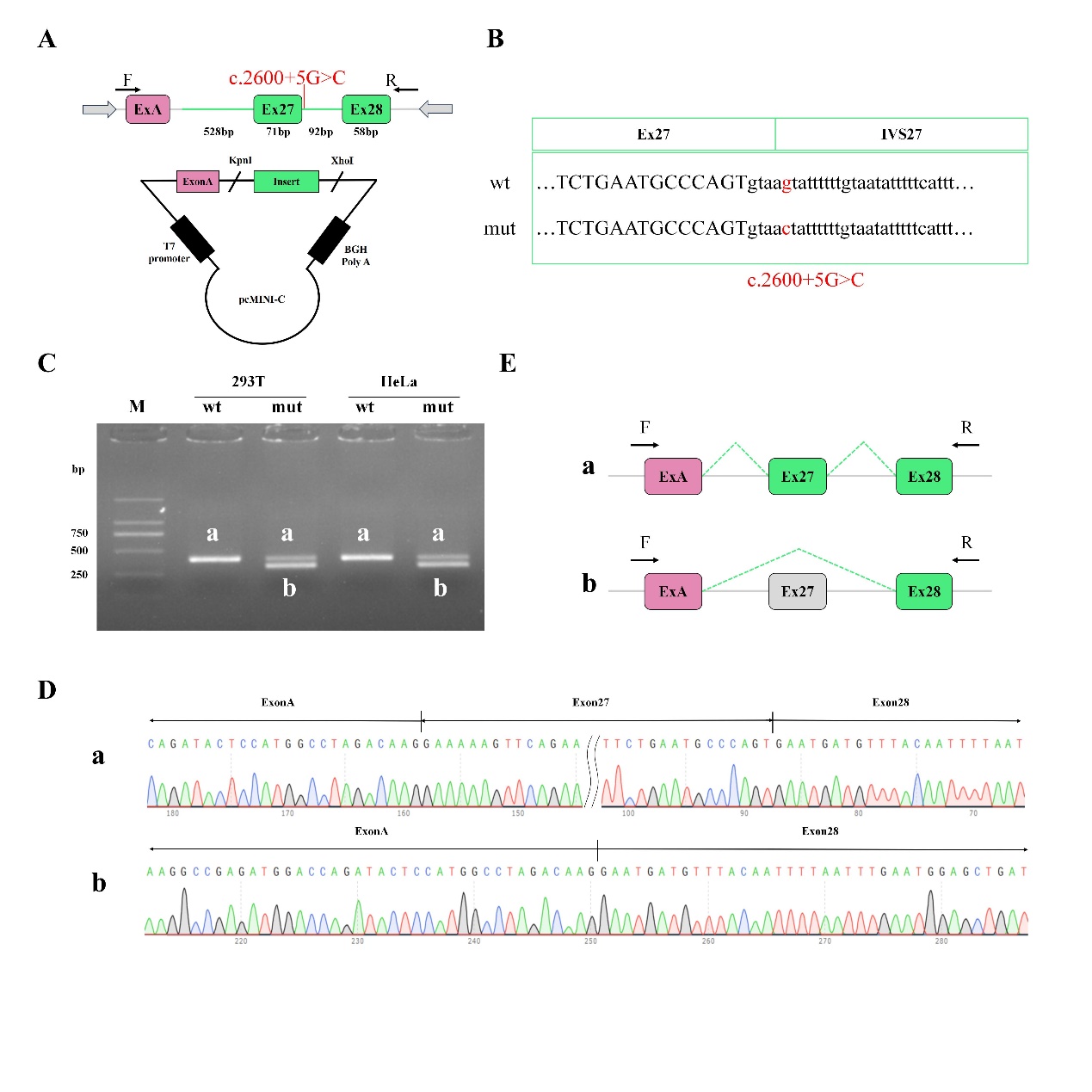


Figure S1 Functional splicing examination of the variant with mini-gene splicing assays by the vector pcMINI-C. **(A)**. Schematic diagram of the constructed mini-gene. ExA were exonic sequence of the plasmid. The green marks were the target sequence **(B)**. Sanger sequencing confirmed that wild-type and mutant fragments were successfully introduced into the mini-gene construct. **(C)**. RT-PCR was performed to verify alternative splicing in the wild-type and mutant groups. Abnormal splicing bands in mutant groups were uncovered in HEK 293T cells and Hela cells. Agarose gel electrophoresis showed that wt had only one band, labeled a and mut produced two bands, labeled a and b. **(D)**. The bands of a and b were identified by Sanger sequencing. PCR product sequencing revealed exon27 skipping. **(E)**. Alternative schematic diagram. wt, wild type; mut, mutant type; M, DL2000 DNA ladder.


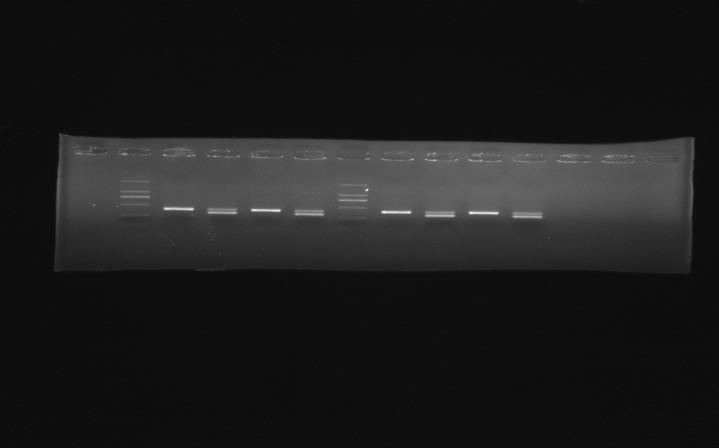


Figure S2 The full uncropped Gels image. RT-PCR was performed to verify alternative splicing in the wild-type and mutant groups. Bands were verified by agarose gel electrophoresis.
